# The Antiviral and Antitumor Effects of Defective Interfering Particles/Genomes and Their Mechanisms

**DOI:** 10.3389/fmicb.2019.01852

**Published:** 2019-08-09

**Authors:** Yicheng Yang, Taibiao Lyu, Runing Zhou, Xiaoen He, Kaiyan Ye, Qian Xie, Li Zhu, Tingting Chen, Chu Shen, Qinghua Wu, Bao Zhang, Wei Zhao

**Affiliations:** ^1^Guangdong Provincial Key Laboratory of Tropical Disease Research, School of Public Health, Southern Medical University, Guangzhou, China; ^2^The First Clinical Medical College, Southern Medical University, Guangzhou, China; ^3^The Second Clinical Medical College, Southern Medical University, Guangzhou, China

**Keywords:** defective interfering particles, defective interfering genomes, interference mechanisms, antiviral effect, antitumor effect

## Abstract

Defective interfering particles (DIPs), derived naturally from viral particles, are not able to replicate on their own. Several studies indicate that DIPs exert antiviral effects via multiple mechanisms. DIPs are able to activate immune responses and suppress virus replication cycles, such as competing for viral replication products, impeding the packaging, release and invasion of viruses. Other studies show that DIPs can be used as a vaccine against viral infection. Moreover, DIPs/DI genomes display antitumor effects by inducing tumor cell apoptosis and promoting dendritic cell maturation. With genetic modified techniques, it is possible to improve its safety against both viruses and tumors. In this review, a comprehensive discussion on the effects exerted by DIPs is provided. We further highlight the clinical significance of DIPs and propose that DIPs can open up a new platform for antiviral and antitumor therapies.

## Introduction

Defective interfering particles (DIPs), caused by the critical absence of part the viral genome, are unable to replicate on their own. They need a standard virus known as the helper virus or complete virus for co-infection. Researchers have discovered that inactive viruses could stop the spread of influenza viruses (Henle and Henle, 1943). Later, von Magnus discovered that the interference in viral replication was caused by incomplete forms of the influenza virus and the incomplete forms proliferated only in the presence of the standard viruses (Von Magnus, 1954). In 1970, the incomplete forms of viruses were renamed DIPs.

Defective interfering particles are of viral origin and have the same structural features as their homologous standard viruses. Typically, a deleted form of the viral genome is called defective interfering (DI) genome (Huang and Baltimore, 1970). DI genomes are truncated forms of the viral genomes generated by most viruses during viral replication. It has been found that DI genomes retain the terminal sequences which are recognized by viral polymerases, the sequences for packaging, a competent initiation site at the 3′end, its complementary sequence at the 5′ end, and a structure or sequence required for encapsulation into a nucleocapsid (Resende et al., 1992; Mura et al., 2017).

The generation of DI genomes is often referred to as the “copy-choice” mechanism, which is caused by the viral polymerase. The polymerase skips from one template to another, or from one part of the template to other parts, and resumes elongation at the 3′ end of the nascent chain synthesized before it further “skips” (Lazzarini et al., 1981; Perrault, 1981). Different types of DI genomes (Kolakofsky, 1976; Leppert et al., 1977) are listed below: (1) Simple internal deletion DI genomes. Deletions are generated when a fragment of the template is skipped (Perrault, 1981; Jaworski and Routh, 2017). (2) Copy-back or panhandle DI genomes (Fodor et al., 1994); The polymerase carries a partially made strand and switches back to transcribe the 5′ end, forming the panhandle shape. (3) Hairpin or snapback DI genomes; The replicase transcribes part of one strand and then uses the new strand as a template, resulting in the formation of a hairpin (Schubert and Lazzarini, 1981). (4) Mosaic or complex DI genomes; various regions may come from the same helper virus genome but in the wrong order, from different helper genome segments, or could include segments of host RNA (Mura et al., 2017). (5) Mutations in DI genome; A novel DI genome type derived from influenza A viruses has been reported. The genomic viral RNA of segment 7 (S7), which encodes proteins and genome packaging signals, carried 37 point mutations affecting the promoter regions (Kupke et al., 2019). Because of the absence or mutation in the critical part of the viral genome, DIPs cannot self-replicate (White and Morris, 1994). They infect cells and interfere with the replication of genetically compatible viruses when co-infected with the standard virus. The standard virus helps to facilitate the replication and packaging of the DI genomes by *trans*-complementation and lead to the release of mainly non-infectious DIPs.

**FIGURE 1:**
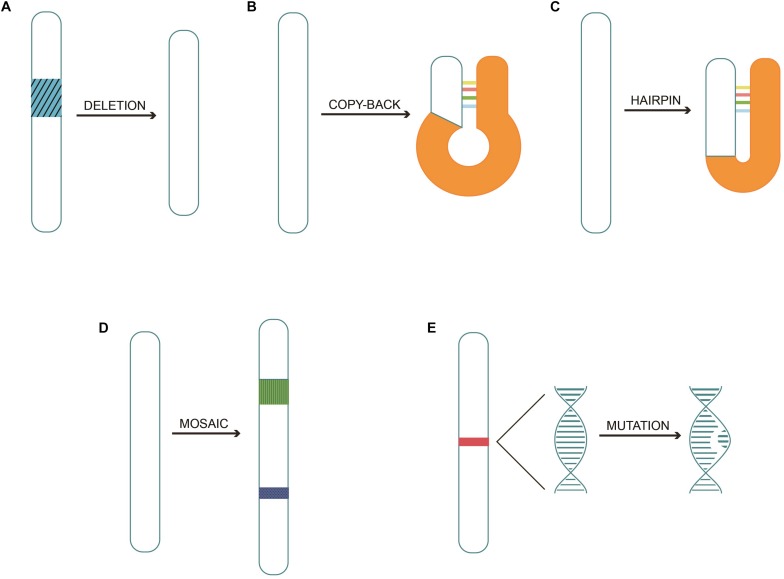
Different types of DI genomes. **(A)** Simple internal deletion DI genomes occurring as part of the template are skipped during replication. **(B)** Copy-back or panhandle DI genomes are generated when the polymerase carrying partially made strand switches transcribe the 5′ end. **(C)** Hairpin or snapback DI genomes are produced when replicase transcribes part of one strand and then uses the new strand as a template strand. **(D)** Mosaic or complex DI genomes combine various gene fragments from the same helper virus in the wrong order, or from different helper viruses, or even from segments of host RNA. **(E)** Mutations DI genomes with a novel DI genome type were discovered recently.

Different types of DI genomes are depicted in Figure 1.

Defective interfering particles are observed during infections of a majority of DNA and RNA viruses. Herpes simplex virus (HSV) (Harty et al., 1993), pseudorabies virus (Wu et al., 1986), geminivirus ageratum yellow vein virus (AYVV) (Stanley et al., 1997), DNA viruses in plants (Huang, 1973; Patil and Dasgupta, 2006), poliovirus (Lundquist et al., 1979), measles (Cattaneo et al., 1988; Pfaller et al., 2015), Sin-dbis virus (Kennedy et al., 1976; Petterson et al., 2016), vesicular stomatitis virus (VSV) (Giachetti and Holland, 1988), influenza virus (Fazekas and Graham, 1954; Kupke et al., 2019), dengue virus (Li and Aaskov, 2014), SARS coronavirus (Raman and Brian, 2005) and West Nile virus (Brinton, 1983) can produce DIPs. Phages are also able to generate DIPs. The serial passage of bacteriophage f1 at high multiplicities of infection results in the appearance of defective deletion mutants (miniphage) and they interfere with the growth of wild-type f1 (Horiuchi, 1983).

For many years in the past, numerous studies have revealed that DIPs exert an antivirus effect and different types of DIPs interfere with viral replication and reproduction through diverse mechanisms. This area is currently a novel research hotspot, however, researchers have pointed out that DIPs cause persistent infection and the virus cannot be removed completely. With the development of gene sequencing and recombination in recent years, DIPs have attracted the interest of researchers again. DI genomes can be probed to increase the security of DIPs.

Meanwhile, DIPs can be constructed to use as an antiviral vaccine and it has also been revealed that DIPs have antitumor effects (Liu et al., 2016). In this review, the interference mechanisms of DIPs and their effects on tumor are summarized. Upon further investigation, DIPs are expected to become a new class of antiviral and antitumor agents with clinical implications.

## The Mechanisms of Interference with Virus

Defective interfering particles interfere with the replication of the parental virus. In addition, cross-interference can be discovered between the closely related VSV-Indiana and VSV-New Jersey viruses (Prevec and Kang, 1970; Schnitzlein and Reichmann, 1976), among different subtypes of the influenza A virus (De and Nayak, 1980), and among different alphaviruses (Weiss and Schlesinger, 1981). Based on studies, the multiple mechanisms of the antiviral effects exerted by DIPs are presented below.

### Stimulating Immune Response

#### Activation of Innate Immune

As a critical part of innate immunity, type I IFNs are thought to be responsible for the antiviral effects when DIPs/DI genomes are derived. Studies showed evidence linking DIPs to type I IFNs (Frensing et al., 2014). A defective influenza A virus RNA (244RNA) served as a protection for mice against a simultaneous challenge of 10 50% lethal doses of the influenza A/WSN (H1N1) virus. The protection from all other subtypes of the influenza A virus was observed. It was indicated that type I IFNs might play a role in protection due to the presence of strong antigens, defective RNAs and virions. The transmission of the preferably packed non-infectious defective virions to nearby cells was also a part of the mechanism (Dimmock et al., 2008). Similar results have been observed for Sendai virus DI genomes. Double-stranded RNA potentially triggers increased expression of type I IFNs (Strahle et al., 2006).

Three pathways contribute to the induction of type I IFNs. Efforts were made to illustrate the underlying mechanisms and pathways. A double-stranded RNA of more than 30 bases in length, appears in the majority of DIPs/DI genomes and is an important inducer of type I IFNs in cells (Marcus and Gaccione, 1989; Strahle et al., 2006). Retinoic acid-inducible gene 1 (RIG-I) serves as a pivotal detector of intracellular RNA and can recognize DIPs/DI genomes with a particular structure (Sanchez et al., 2016; Vasou et al., 2017). The cytoplasmic DI genome, rather than full-length virus genome in the Sendai virus, was preferentially bound with RIG to exert immunostimulatory activity. In influenza-infected cells, RIG-I, the intracellular RNA detectors, preferentially bound to shorter genomes, including the DI genome. An immunostimulatory activity test confirmed that the downstream signal of RIG-I induced the expression of type I IFNs (Baum et al., 2010). Other studies also suggest a RIG-I role in the induction of type I IFNs. DI RNAs, especially in copy-back form, which are types of pathogen associated molecular patterns (PAMPs) with stem-loop structures, are the ideal ligands for RIG-I and have strong inducibility of type I interferons (IFNs) (Yoshida et al., 2018). RIG-I recognition of RNAs with 5′-triphosphates or 5-diphosphates effectively augments the production of IFNs (Goubau et al., 2014), A DI RNA containing both 5′-triphosphates and an intermediate-length of the double-stranded region found in the measles virus is the perfect agonists for RIG-I. Quantitative real-time RT-PCR results revealed an immunostimulatory activity characterized by increased expression of type I IFNs, which is also significant for the subsequent induction of adaptive immunity (Ho et al., 2016). Thus, sensing of cytoplasmic RNA, RIG-I is triggered to deliver downstream signals by interactions between the helicase and adaptor protein IFN-β promoter stimulator 1, resulting in an innate immune response mediated by type I IFNs (Mogensen, 2009). Previously considered as a negative modulator, LGP2 binds to DIPs/DI genomes and up-regulates type I IFNs expression. LGP2 belongs to the cytoplasmic pattern recognition receptor as RIG-I but is the absence of a C7ARD domain required to trigger the IFNs signaling cascade via mitochondrial antiviral signaling (MAVS) activation. DI genomes from a recombinant measles virus were a strong inducer of type I IFNs after 24 h infection *in vitro*. In addition, a comparison of binding efficacies during infection of the virus and its DI genomes indicated that measles virus DI RNAs appeared to enrich on LGP2, highlighting the crucial role of LGP2 in type I IFNs mediated response (Mura et al., 2017). Melanoma differentiation-associated protein 5 (MDA5) was associated with type I IFNs regulation (Yount et al., 2008; Tapia et al., 2013; Sun et al., 2015). MDA5 is a positive modulator in the cytoplasm but differs from RIG-I in distinct recognition patterns. The study showed that measles DI particles could be an agonist for MDA5 though the fraction magnitudes low relatively (Runge et al., 2014; Mura et al., 2017). It could be attributed to the short structure of the measles virus DI particles and a deep sequencing approach may be needed to elucidate the connection between MDA5 and DI particles. Upon activation, MDA5 induces IFNs expression, which is similar to the RIG-I activity, in exerting an antiviral response.

Activation of RIG-I, LGP2, and MDA5 leads to the upregulation of type I IFNs and changes in the immune environment. However, the characteristics of different structures of DIPs/DI genomes are known to associate with different pathways. For example, the copy-back DI genome is more efficient in binding to RIG-I. Genome length is another aspect that affects binding. DI genomes with short double-strands can be detected by RIG-I, whereas long double-stranded RNA without phosphate is preferably bound to MDA5. Last but not least, 5′-triphosphate RNA of DI particles, especially those rich in U residues, contribute to RIG-I activation, however, little is known about single-stranded DI genomes in stimulating type I IFNs expression. The relation between host cellular pathways and antiviral outcomes of type I IFNs remains unclear. Therefore, attention should be paid to elucidating the regulation of cellular pathways during viral infection with the DI genome. As the virus can produce various type of genomes, different pathways may result in a different effect. In addition, the influence of the secondary structure of viral RNA in activation is also open and worth investigating as viral RNA varies highly. Addressing the problems could shed light on technique improvement when it comes to obtain optimal DIPs/DI genomes with gene modification.

Recent studies involving animal models achieved some advancements. DIPs/DI genomes show protection not only from the homologous virus but also from the heterologous virus. A study (Scott et al., 2011) demonstrated that DIPs of influenza virus Type A showed heterologous protection to influenza virus Type B, and DIPs stimulated the production of type I IFNs. The protective effect of DIPs against influenza virus Type B was reduced after knocking out the type I IFNs receptor gene in mice, suggesting that the production of type I IFNs was significant. It was indicated type I IFNs involved in the alteration of the antiviral environment in immune cells or modulation of the immune system, which might generate the additional heterologous protective effect. Furthermore, influenza A and B viruses do not interact with each other when they replicate (Scott et al., 2011). However, intranasal administration of the DIPs of influenza virus Type A helps to fight pneumonia viruses besides influenza A and B viruses of mice (Easton et al., 2011). The underlying mechanisms of heterologous protection are unknown. It could be hypothesized that type I IFNs play a role by increasing the activity of natural killer cells. Type I IFNs is a broad-spectrum antiviral protein and effective immune modulator. Adaptive immunity could be another potential factor to eliminate heterologous virus because immune cells and their functions linking innate immunity and adaptive immunity are greatly enhanced by type I IFNs (Mellman and Steinman, 2001). Therefore, the induction of type I IFNs, upon strong stimulation of DIPs/DI genomes, activates the immune system and heterologous protection.

In the Sendai and respiratory syncytial virus infections, defective viral genomes can facilitate viral persistence by promoting TNFR2/TRAF1-dependent mechanism and stimulating MAVS-mediated TNF response (Xu et al., 2017). Thus, the mechanism employed by DIPs to influence the innate immune remains uncertain as there are opposing functions of DIPs in common viruses.

#### Activation of Adaptive Immune

Defective particles/genomes can trigger the maturation of dendritic cells (DCs) *in vivo* and enhance antigen-specific immunity to viruses in the host cells (Mercado-Lopez et al., 2013; Frensing et al., 2014). The mechanisms are not clearly understood and are worth investigating. Peripheral antigen present cells, including DCs, may have a high probability to contact with preferably packed DIPs/DI genomes, which are strongly antigenic but non-infectious. Type I IFNs induction improves the ability of DCs to efficiently bind agonists and process antigen, as well as to up-regulate cytokines, linking innate immunity to adaptive immunity. In addition, it is speculated that production of antibodies exerts antiviral activity and that the expression of type II IFN, also known as IFN-γ, following DCs maturation, is involved in inducing specifically antiviral T cells *in vivo* (Mercado-Lopez et al., 2013).

Dendritic cells maturation is essential in the activation of adaptive immune responses that maintain long-lasting protection against reinfection with the same virus. DIPs of Sendai virus provide higher titer of a DC-activating virus replication intermediate product, most probably dsRNA, and it is recognized as pathogen-associated stimuli to trigger the TLR-independent pathway. Costimulatory molecules, chemokines, chemokine receptors, and numerous pro-inflammatory cytokines (Mellman and Steinman, 2001; Yount et al., 2006, 2008). Interestingly, the DCs maturation ability by the DI particles was further improved and demonstrated when DI particles were added into a virus that weakly activates DCs. It could be hypothesized that increased levels of anti-genomic promoter copy-back DIPs and other viral compositions existing in the cell membrane or in endosomal compartments could trigger the DCs maturation genes, thus initiating the type I IFN signaling-dependent and -independent DCs maturation. Thus, the effects of DIPs-induced DC maturation involved in the antiviral mechanisms which could be rational in designing viral vaccines.

With the outstanding antiviral effect (Wasik et al., 2018), DIPs are expected to be used in vaccines or its adjuvants to stimulate an adaptive immune response. A study has reported that the DIPs of influenza virus Type A (the 244 DI virus) could protect Ferrets Mustela Putorius furo (Dimmock et al., 2012) from being infected by the complete influenza virus particles and alleviate the symptoms. The vaccination of mixed intact influenza virus and DIPs induced high titer hemagglutinin-inhibiting antibodies in ferrets. The intact Influenza virus could not be detected after 8 days of vaccination. Moreover, the DI244 virus could also not be detected after 10 days, suggesting that a high titer hemagglutinin inhibitory antibody also kills DIPs. Evidence confirmed DIPs/DI genomes are involved in antibody induction and T cell activation. The DI244 virus exerted an anti-influenza virus effect in human respiratory cells (Smith et al., 2016). Simultaneous vaccination of herpes virus type 2 and a recombinant HSV type 2 glycoprotein D (gD2) induced, high titers of neutralizing antibodies, and HSV-specific CD8 + T cell responses (Dudek and Knipe, 2006). As mentioned above, activated adaptive immune can inhibit heterologous viruses that express similar structural antigens as the standard virus. Therefore, DIPs can be highly promising candidates for vaccine development.

The properties of DIPs/DI genomes, which are non-infectious and decent antigens, attracted the attention of researchers who later utilized the gene recombination technology to modify DIPs as a safe vaccination. Adenoviral vectors induced long-lasting and effective T cell responses against the hepatitis C virus and were thought to be a novel vaccine virus (Wen et al., 2013; Dimmock and Easton, 2014). When rhesus monkeys were treated with DIPs of replication-defective hepatitis C virus and the adenovirus recombinant DIPs, the cellular immune effects and antibody responses against multiple viruses were induced in rhesus monkeys. This study provides a rationale for the development of other antiviral vaccines and clinical trials. In addition, it was found that DIPs of the mumps virus could alleviate the neurotoxicity of the wild-type mumps virus, providing a basis for new mumps vaccines (Santak et al., 2015). Some researchers constructed two adenoviral interfering particles with replication-related genes deletion, and they were expressed on the surface by highly recombinant fusion proteins and hemagglutinin. After being injected into mice, these DIPs induced antibody production, along with CD4 + and CD8 + T cell responses specific to the peste des petits ruminants virus (PPRV) antigen (Rojas et al., 2014). DIPs/DI genomes are a perfect candidate for vaccination and worth developing as a safe agent against the virus. Deep gene sequencing and gene modification shed light on a prospective future for scientists who could create DIPs/DI genome vaccine without pathogenicity, whereas, the evaluation to confirm if modified DIPs/DI genomes lead to multiple frequencies of virus mutations and if these mutations will result in adverse outcomes, remains ongoing.

**FIGURE 2:**
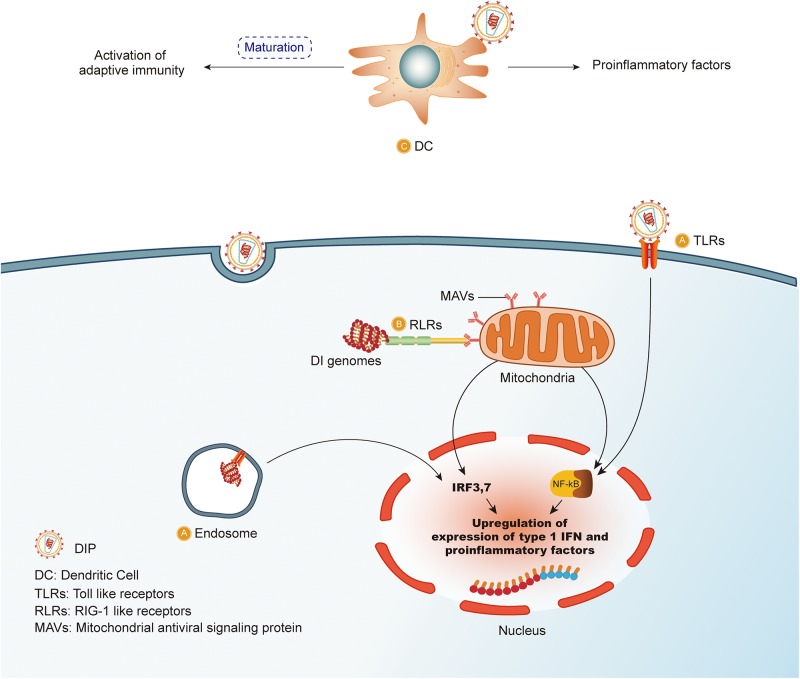
DIPs stimulate immune system. **(A)** Defective interfering particles (DIPs) activate the downstream NF-κB pathway and IRF3,7 release by binding to TLRs on the cell membrane or the surface of the endosomal membrane, resulting in the release of IFN-1 and other pro-inflammatory factors against the virus. **(B)** DIPs activate RLRs and increase the release of pro-inflammatory factors through the above pathways. **(C)** DCs *in vivo* recognize DIPs, which trigger maturation of the DCs, consequently activating the immune system.

Thus far, DIPs/DI genomes provide promising immune stimulatory effects, and comprehensive pre-clinical data are available for the DIPs of the influenza virus Type A (Dimmock and Easton, 2014), Sendai virus (Yount et al., 2008) and Semliki forest virus (Barrett and Dimmock, 1986). However, there are a few key aspects to carefully note. Although the majority of studies suggest that administration of DIPs/DI genomes have an inhibitory or even therapeutic effect on the standard virus, it is noticeable that the replication process is reduced, but not abolished. Thus, a deeper understanding of the mechanisms is needed in order to clear up the standard virus when it comes to clinical use. In addition, the persistence of influenza A virus RNAs was reported in cultured cells under conditions where the virus was not replicating (Marcus and Sekellick, 1977; Cane et al., 1987; Cane and Dimmock, 1990), thereby indicating an urgent need to further investigate the mechanisms and elimination measures of the persisted DIPs/DI genomes as DIPs/DI genomes have a promising future as an antiviral agent. Alternatively, it could lead to an adverse outcome when new infections happen. Therefore, pre-clinical trials are required to support the potential use of DIPs as antiviral agents. The multiple mechanisms by which DIPs may activate the immune system are depicted in Figure 2.

### Suppressing Virus Replication Cycle

The typical virus replication cycle contains six stages including adsorption, penetration, uncoating, replication, packaging, and release (Yin and Redovich, 2018). DIPs or DI genomes exert an antiviral effect by suppressing different stages of the virus replication cycle.

#### Blockage of Adsorption and Penetration

Most DIPs can attach to cells unless a surface protein required for entry is absent, which prevents viruses from invading cells. After DIPs invade cells, deletion of genomic fragments affects the interaction between viruses and receptors on the cell membrane.

Defective interfering particles have been implicated as cofactors in reducing the prevalence of dengue virus (Aaskov et al., 2006) as well as the severity of the disease (Li et al., 2011). A defective dengue virus type 2 genome was found in persistently infected mosquito C6/36 cells. In the infected Aedes albopictus C6/36 cells, it was found that deletion of the dengue virus type 2 genome, which encodes the E protein, blocked the infectious virus from adsorption and penetration, while those lacking the NS5 gene showed inhibited replication and methylation (Juarez-Martinez et al., 2013). However, the specific mechanism by which DIPs/DI genomes block the complete virus from adsorption and penetration needs to be further explored, and whether this phenomenon exists in DIPs/DI genomes of other viruses also needs to be further observed.

#### Interference With Replication

Defective interfering particles compete for essentials which are synthesized by the helper virus or host cells in limited amounts, which results in inhibition of the replication of the standard virus (Dimmock and Easton, 2015). It is reported that the influenza virus DIPs interfered with the replication steps rather than the transcription steps of viral RNA synthesis (Nayak et al., 1978). DIPs of VSV, whose RNA genome encodes five proteins: the nucleocapsid (N) protein that encapsulates the genomic RNA, phosphoprotein (P) and large (L) protein which make up the polymerase, glycoprotein (G) involved in cell-surface binding, and matrix (M) protein important both for virion formation and inhibition of host antiviral gene expression (Hanke et al., 2017), interfered with the replication of helper virus (Palma et al., 1974).

Deletion forms of DIPs contain a smaller genome than the helper virus. The DI genome can therefore replicate faster than the standard genome, and the polymerases can synthesize more copies of the DI genome in a certain period in comparison with the standard genome until the DI genome predominates. After several rounds of replication, the number of the DI genome easily exceed that of the helper genome, even if the ratio of DI: helper was initially very low (Marriott and Dimmock, 2010). DIP can be recognized as a molecular parasite, and due to the existence of a high amount of DI genomes, some negative factors are sequestered by DI genomes, such as viral polymerase components or host proteins (Roux et al., 1991). For example, studies have shown that DI and standard genomes compete with a soluble extract containing the viral L and P proteins, i.e., the components of the viral RNA-dependent RNA polymerase. DIPs of influenza virus type A compete for polymerases with the helper virus. As more DIPs are produced, a large number of enzymes and other resources would be exhausted to ultimately cause the inhibition of the helper virus (Laske et al., 2016). DI RNA also interferes with the replication (Davis and Nayak, 1979). Li et al. (2011) determined that DI dengue viral particles replicated in the same manner as a full- length genome. The replication of both DI and full-length genomes needs the structure of the 5′ and 3′ regions of the viral genome. As DI genomes lack the 5′ and 3′ regions of the genome and the RNA encodes for no protein, they are completely dependent on wild type viruses to make up for this deficit, and consequently, they compete with functional genomes for viral and host cell proteins. Additionally, defective deletion mutants of bacteriophage also interfere with the growth and replication of wild-type f1 (Horiuchi, 1983).

#### Effect on Packaging

Several studies have shown DIPs/genomes exerted an antiviral effect by affecting the packaging steps besides the RNA synthesis step of the standard virus. The particles of viruses, or virions, which contain a gene 1-derived DI RNAs, were selectively depleted for the full-length gene 1 of a complete H7N7 influenza virus, revealed using northern blot analysis. The study convincingly showed that the expression of gene 1 in the infected cells did not dramatically reduce but the amount of the “parent” segment in the released virus was greatly diminished, illustrating a competitive effect on the packaging (Duhaut and McCauley, 1996).

Dengue viral DIPs and influenza viral DIPs, whose genomes contained deletions, could also affect the process of packaging besides RNA synthesis by viral polymerase (Duhaut and McCauley, 1996; Li and Aaskov, 2014). The abundant and smaller DI genomes were more likely to be packaged into new virus particles, and they have a faster rate of packaging than standard virus genomes (Liao et al., 2016). It was found that cloning of influenza A DI RNA (1/244) played an interference role by replacing the cognate full-length segment-1 RNA in progeny virions, and therefore, cloned influenza A DI RNA was preferentially packaged (Meng et al., 2017). Mutation forms of DIPs are also able to affect the packaging process. An unknown, novel DIPs of Influenza virus type A, OP7 virus contained numerous point mutations instead of large deletions and was defined by researchers using single-cell analysis. Generated by naturally genetic substitutions due to point mutations, the OP7 virus was observed to reduce the hemagglutination assay (HA) titers in a human cell line when co-infected with a standard virus besides a severe reduction in the infectivity of the released virions. Promisingly, protection of other subtypes of influenza virus type A in Madin-Darby canine kidney (MDCK) cells, such as the pandemic influenza virus A/California/7/2009 of H1N1 subtype (H1N1-pdm09) and the H3N2 subtype influenza virus A/Hong Kong/4801/2014, was reported. The interference could be accounted for by the advanced genome replication, and more importantly, preferential packaging of DIPs. They found that the particular type of DIPs produced an excessively higher quantity of S7 vRNA (intracellularly and in the released virus particles), which played a role in promoter regions, encoded proteins and genome packaging signals. OP7 was considered non-infectious; thus, the infection of Influenza virus was partially suppressed in these experiments (Kupke et al., 2019).

#### Influence on Release

The cellular endosomal sorting complexes required for transport (ESCRT) is composed of cytosolic protein complexes. With a few accessory proteins, these ESCRT complexes enable a unique mode of membrane remodeling that allows membranes to bend or bud away from the cytoplasm (Carlton and Martin-Serrano, 2007; Babst, 2011; Schmidt and Teis, 2012). The release of viral particles, which is also called viral budding, releases the virions from cells via the hijacking of host cell ESCRT machinery (Jouvenet et al., 2011; Votteler and Sundquist, 2013). It was found that the DIPs produced by the lymphocytic choriomeningitis virus inhibited the release of homologous viruses and promoted DIPs release by modifying the ESCRT pathway, thereby exerting an antiviral effect (Ziegler et al., 2016). OP7 viruses (as mentioned above) were reported to adversely affect the release step of the standard virus.

Although numerous studies indicate that DIPs can suppress different stages during the virus replication cycle, it still lacks data for this dynamic intracellular behavior.

**FIGURE 3:**
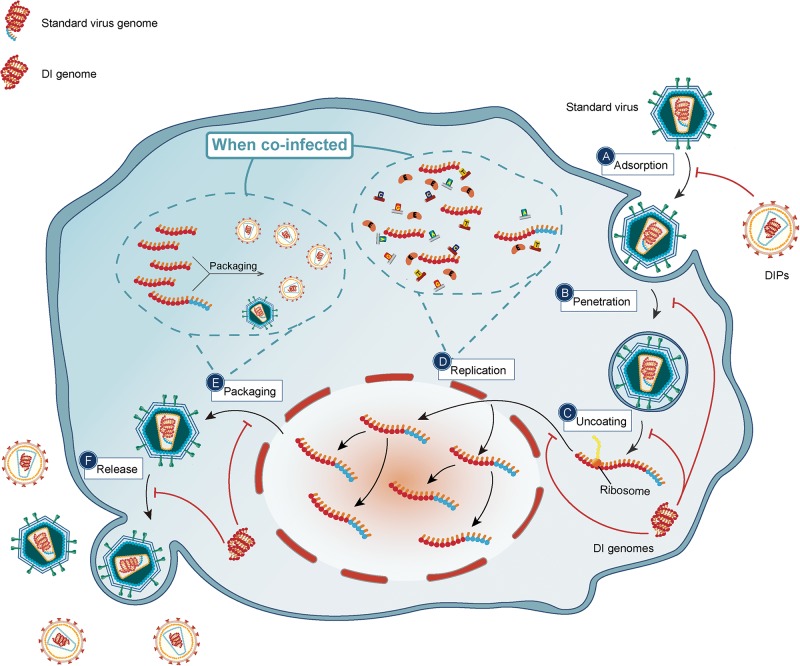
DIPs/DI genomes suppress the cycle of standard viruses. DIPs/DI genomes have deletion in critical parts compared to standard viruses. The red arrows represent the inhibitory effect of DIPs. By interaction with immune system, the life cycle of standard viruses including **(A)** adsorption; **(B)** penetration; **(C)** uncoating; **(D)** replication; **(E)** packaging; **(F)** release is under suppression. With relatively shorter genes, DI genomes are able to replicate more copies in per unit time and outnumbered DI genomes will compete for packaging materials, and thus DIPs/DI genomes interfere with replication and packaging of standard viruses.

The multiple mechanisms by which DIPs may suppress with virus replication cycles are depicted in Figure 3.

We summarized the different mechanisms by which DIPs interfere with virus replication (see Table 1).

**TABLE 1:** Summary of mechanisms for DIPs to interfere with virus replication.

**Mechanisms**	**Viruses DIPs**	**DI genomes forms**	**Gene**	**References**
*Stimulating immune response*	Influenza virus	Simple internal deletions DI genomes;	-ssRNA	Dimmock and Easton, 2014; Vasou et al., 2017
	Respiratory syncytial virus	Simple internal deletions DI genomes; Copy-back DI genomes	-ssRNA	Borchers et al., 2013; Xu et al., 2017
	Ebola virus	/	-ssRNA	Stanley et al., 2014
	Rift valley fever virus	Mutations DI genome	-ssRNA	Bouloy et al., 2001
	Measles	Copy-back DI genomes	-ssRNA	Mura et al., 2017
	Semliki forest virus	Simple internal deletions DI genomes	+ ssRNA	Barrett and Dimmock, 1986; Puglia et al., 2013
	Rotavirus	/	dsRNA	McNulty et al., 1978
	Sendai virus	Copy-back DI genomes	dsRNA	Mellman and Steinman, 2001; Yount et al., 2006; Yount et al., 2008; Yoshida et al., 2018
*Suppress virus replication cycle*	Vesicular stomatitis virus	Simple internal deletions DI genome	-ssRNA	Palma et al., 1974; Hanke et al., 2017
	Influenza virus	Simple internal deletions DI genome; Mutations DI genome	-ssRNA	Nayak et al., 1978
	Mumps virus	Simple internal deletions DI genomes	-ssRNA	Santak et al., 2015
	Rabies virus	/	-ssRNA	Morimoto et al., 2005
	Poliovirus	Simple internal deletions DI genome; Mutations DI genome	+ ssRNA	Lundquist et al., 1979
	St. Louis encephalitis virus	Simple internal deletions DI genome	+ ssRNA	Salas-Benito and De Nova-Ocampo, 2015
	Epidemic encephalitis virus	Simple internal deletions DI genome	+ ssRNA	Park et al., 2013
	Sindbis virus	Simple internal deletions DI genomes	+ ssRNA	Poirier et al., 2015; Kebschull et al., 2016
	Hepatitis A virus	/	+ ssRNA	Siegl, 1992
	West Nile virus	/	+ ssRNA	Brinton and Fernandez, 1983
	Coxsackie virus	/	+ ssRNA	M’Hadheb-Gharbi et al., 2007
	Rubella virus	/	+ ssRNA	Tzeng et al., 2001
	Dengue virus	Simple internal deletions DI genomes	+ ssRNA	Aaskov et al., 2006; Li et al., 2011; Juarez-Martinez et al., 2013
	Yellow fever virus	Simple internal deletions DI genomes	+ ssRNA	Juarez-Martinez et al., 2013
	Sand virus	/		Ziegler et al., 2016
	Herpes virus	Simple internal deletions DI genomes;	dsDNA	Charvat et al., 2012
	Adenovirus	/	dsDNA	Remenick et al., 1988

## DI Constructs Show an Anticancer Effect

Many studies have shown the antitumor effects of defective viruses. After ultraviolet irradiation, a particle of the Sendai virus that was replication-defective but still retained the original protein structure was obtained. These replication-defective particles could suppress the growth of CT26 colon cancer cells *in vitro* and prolong the survival of mice (Shi et al., 2013). Such particles also exhibited a pro-apoptotic effect on the human breast cancer cell line, MDA-MB-231, through the endogenous mitochondria and exogenous death receptor pathway (Chen et al., 2014). Consistently, several studies have discovered the inhibitory effect of replication-defective viruses on melanoma (Zhang et al., 2012, 2014) and hormone-resistant prostate cancer (Kawaguchi et al., 2009).

An enthusiastic interest was further developed in the therapeutic potential of DI constructs and their use in immunotherapy and oncogenesis because of the higher specificity and effectiveness. For example, DIPs of a hemagglutinating virus of Japan (HVJ) can promote more significant DC maturation and type-I IFNs production to kill cancer cells than other defective viruses (Yount et al., 2006; Liu et al., 2016). Researchers are now focusing on the antitumor effect exerted by DIPs and DI genomes.

### DIPs/DI Genomes Induce Cancer Cell-Selective Apoptosis

Defective interfering particles/defective interfering genomes induce cell-selective apoptosis in cancers through RIG-I downstream signaling (Horiuchi, 1983; Ho et al., 2016; Mura et al., 2017). RIG-I preferentially recognizes short RNA sequences (∼300 bp to 1 kb) with 50 triphosphoric (50 ppp) ends (Kato et al., 2008; Hwang et al., 2012) and is thought to be one of the RNA sensing pattern recognition receptors (PRRs). PRRs are another critical costimulatory molecule of immune cells, notably myeloid cells (macrophages and DCs) (Takeuchi and Akira, 2010). PRRs agonists are able to activate phagocytosis and antigen presentation by myeloid cells residing in the tumor micro-environment. In pre-clinical trials, PRR agonists can also directly induce immunogenic cell death, a particular type of apoptosis that triggers immune responses specific for dead cell-associated antigens (Shekarian et al., 2017). It is a new way to use DIPs/DI genomes as PRR agonists to exert an antitumor effect.

Research has suggested that certain Sendai virus DIPs, which contain a short RNA genome (∼550 nucleotides), can strongly induce RIG-I-dependent apoptosis. The copy-back type of DIPs that exist in the Cantell strain of HVJ more effectively activates the pro-apoptotic genes in cancer cells and induces more apoptotic effects than the Sendai/52 strain or Cantell strain, which contains fewer DI particles. In the xenograft mouse model, intra-tumor transduction of DI particle-derived RNA synthesized by *in vitro* transcription (*in vitro* transcribed (IVT)-B2) significantly reduces the size of human prostate cancer. With a double-stranded region in its secondary structure and 5′-triphosphate, IVT-B2 can trigger RIG-I downstream signaling, which is associated with the activation of apoptotic genes [including NOXA and/or TNF-related apoptosis-inducing ligand (TRAIL)], increase in type-1 IFNs production, and activation of pro-apoptotic genes in cancer cells (Liu et al., 2016). Thus, such PRRs agonists, DI genomes or other analogous reagents that induce RIG-related anti-cancer effects, along with other therapeutic drugs, are able to enhance the antitumor effect of the immune system, and as a consequence, cancer cell growth could be suppressed.

Further, cancer cells are susceptible to RIG-I induced apoptosis (Wu et al., 2017). In the study, the underlying mechanism of activation of RIG-I-dependent apoptosis in cancer cells is more likely due to binding of the DIP RNA to RNA sensing PRRs during viral infection, which leads to the activation of signaling proteins, tumor necrosis factor receptor-associated factor (TRAF), and serine kinases. Such kinases lead to the activation of master transcription factors, such as interferon regulatory factor 3/7(IRF3/IRF7), and NF-kB signaling.

However, few research data propose the antitumor effect of DIPs/DI genomes, and more in-depth studies are needed to address the relation between different cancer cells and DIPs/DI genomes. In the study mentioned above, the copy-back type of DIPs played an antitumor role through activation of the RIG-I downstream signaling pathway. However, we need to further explore whether other DIP types can inhibit tumor cells and what mechanisms they employ for their antitumor effects.

### Activation of Dendritic Cells and T Cell-Induced Immunity

In experiments with mice, the DIPs of the Sendai virus exerted an antitumor effect by activating antitumor immunity, which is induced by DCs and T cells. The experiment indicated that in mice administrated with DIP, the tumor growth is inhibited and the tumor volume is significantly reduced (Shi et al., 2014; Liu et al., 2016). In these experimental mice, more mature DCs were observed in the tumor tissues, along with an increase in the release of cytokines as well as the expression levels of CD4, CD8, and CD11C mRNA. Flow cytometry analysis showed that the expression of markers of DCs maturation, including CD40, CD80, and CD86, is dose-dependently increased by DIPs. Thus, the antitumor effects of DIPs are related to the activation of DCs and T cell-induced immunity.

Studies using mice with severe combined immunodeficiency suggest that natural killer (NK) cells are also involved in the anti-cancer effect, which is induced by IVT-B2-RNA. After the administration of IVT-B2 RNA, active NK cells might be attracted to the tumor via chemoattractant CXCL10, and the IFN-β might enhance NK cell activity in the tumor microenvironment (Liu et al., 2016). Thus, DCs display more antigens of tumor cells to exert the antitumor effect, and mature DCs express more co-stimulating factors, which is essential to activate immune cells and tumor killing effects (Takeuchi and Akira, 2010).

**FIGURE 4:**
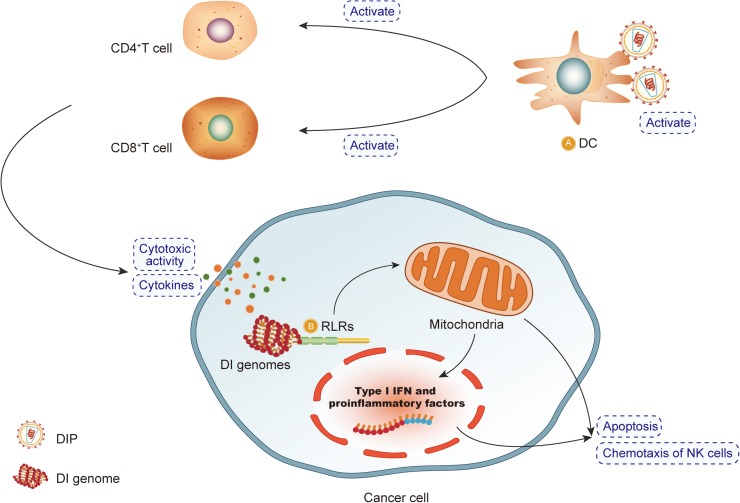
DIPs exert an antitumor effect. **(A)** Defective interfering particles (DIPs) activate DCs, CD4 + T cells and CD8 + T cells to produce cytokines. DIPs stimulate the immune system to play an antitumor effect. **(B)** After recognizing RIG-like receptors, DIPs activate the apoptosis pathway to promote the apoptosis of cancer cells.

The mechanisms by which DIPs exert an antitumor effect are depicted in Figure 4.

## Conclusion

The rapid development of genome sequencing helps researchers design DI genomes for therapy against viruses and tumors. The antiviral and antitumor effects and safety evaluation of DIPs are still in the pre-clinical trial stage, but there are many difficulties to overcome. For instance, DIPs may lead to the drug resistance of standard viruses, to traditional antiviral drugs, which could cause the spread of viruses. Further, the diversity of defective fragments of DIPs severely affects their production and efficacy, and the dose of DIPs required for treatment is difficult to determine. Moreover, DIPs may show different effects *in vitro* and *in vivo*. With current genome sequencing technologies (Beauclair et al., 2018), DIPs can be produced with the same genomic sequence as naturally occurring DIPs, indicating the possibility for clinical application and production.

So far, many studies have confirmed that DIPs interfere with the reproduction of viruses. In this review, we provide a comprehensive summary of different mechanisms by which DIPs may exert antiviral effects. Experimental studies have reported the antitumor ability of DIPs, which could be used as vaccines. Nowadays, although various therapies are explored to treat cancer, including surgery, chemotherapy, radiotherapy and immunotherapy, these treatments may induce severe side effects and/or drug resistance. DIPs are redeemed as being a possibly more efficient way to eliminate cancer cells with minimal side effects. Scientists have built an experimental and computational framework for detecting DIPs-associated deletions in the influenza A and B virus, using deep genome sequencing approaches and the precise mechanisms underlying the regulation of DIPs/DI genomes (Alnaji et al., 2019). Reports show that it is now possible to generate pure production of DIPs/DI genomes in the absence of standard virus *in vitro* (Bdeir et al., 2019; Yamagata et al., 2019). These techniques shed light on the further development of DIPs/DI genome because of the improved safety in their use. With advanced gene sequencing and genetic recombination technology, modified DIPs can be constructed to exert antiviral and antitumor effects. In addition, compared with spontaneously derived DIPs, side effects, such as continuous infection of the virus, are significantly reduced in modified DIPs. Through further in-depth research, DIPs are expected to become a new class of antiviral and antitumor agents for clinical implications.

## Author Contributions

WZ, BZ, QW, CS, and LZ were involved in the conception and writing of the manuscript. YY, TL, RZ, and XH wrote the manuscript. KY, TC, and QX prepared the figures and tables. WZ and BZ checked the manuscript. All authors read and approved the manuscript.

## Conflict of Interest Statement

The authors declare that the research was conducted in the absence of any commercial or financial relationships that could be construed as a potential conflict of interest.
